# Chemotherapy-induced necrotising tumour lysis and perforation of a huge gastric cancer simulating emphysematous pancreatitis

**DOI:** 10.3332/ecancer.2020.1054

**Published:** 2020-06-11

**Authors:** Frank S Fan, Chung-Fan Yang

**Affiliations:** 1 Section of Haematology and Oncology, Department of Medicine, Ministry of Health and Welfare Changhua Hospital, Chang-Hua County, Taiwan; 2 Department of Pathology, Ministry of Health and Welfare Changhua Hospital, Chang-Hua County, Taiwan; a https://orcid.org/0000-0002-8123-6941; b https://orcid.org/0000-0002-7366-4380

**Keywords:** gastric cancer, chemotherapy, dihydropyrimidine dehydrogenase, Bcl-2, emphysematous pancreatitis

## Abstract

A 56-year-old man was diagnosed to have a huge gastric cancer extending from the lesser curvature of the stomach to the pancreas with multiple hepatic and peritoneal metastases. Two days after completing chemotherapy with cisplatin plus high dose leucovorin and fluorouracil, drastic necrotising tumour lysis led to gastric perforation and septic shock most likely due to bacterial peritonitis. The image of tumour lysis looked like an emphysematous pancreatitis. Afterwards, immunohistochemical study of the tumour specimen confirmed moderate positivity of dihydropyrimidine dehydrogenase and absence of Bcl-2 expression. The incomplete expression of dihydropyrimidine dehydrogenase and total deficiency of Bcl-2 are considered to be the main underlying causes of such extraordinary chemosensitivity and so severe a tumour lysis phenomenon. Pre-emptive intensive survey of possible biomarkers of chemosensitivity is thus highly recommended upon treating a massive gastric cancer.

## Introduction

Spontaneous perforation of gastric cancer is occasionally seen with an incidence ranging from 0.39% to 9.6% of all gastric cancer patients as reported by different groups [1–4]. The outcome after emergency surgery in patients with free perforation depends on the stage and whether a curative resection could be performed [5], but is poorer than that in T3 tumour patients without perforation [6].

Although modern neoadjuvant chemotherapy makes conversion gastrectomy possible and improves survival in advanced gastric cancer patients [7] with even an exciting case report of pathological complete remission [8], gastric perforation caused by chemotherapy or sometimes chemotherapy combined with targeted therapy remains a complication incidentally seen [9–11].

We present our unusual experience in treating a patient with dramatic gastric perforation and septic peritonitis resulting from severe necrotising lysis of his massive gastric tumour shortly after systemic chemotherapy. The clinical picture and image study looked very much like an emphysematous pancreatitis.

## Case presentation

A 56-year-old acute ill-looking man came to our emergency unit with the chief complaint of hiccups for four days in June 2019. General weakness, abdomen fullness, epigastric discomfort and poor intake had been noted for several weeks prior to this visit. The patient was not sure whether there was tarry stool but his daughter observed a certain weight loss of the patient. In accordance with that, his body mass index was 18.4 and his body surface area was 1.5 square meters at presentation. He did not have other major systemic diseases except a long history of type 2 diabetes mellitus with irregular control. Alcohol abuse was denied. The physical examination revealed a soft abdomen with tenderness over epigastrium and upper left quadrant. There was no rebounding pain and palpable mass.

Laboratory tests disclosed an elevated serum alkaline phosphatase level of 420 iu/L (normal 32~91) and normocytic anaemia with white cell count 9,300/mL, haemoglobin 10.6 g/dl, mean corpuscular volume 84.7 fl, platelet count 282,000/mL, segments 81.5%, lymphocytes 9.3%, and monocyte 7.6%. There were no active lung lesions in the chest X-ray routine film.

**Figure 1.**
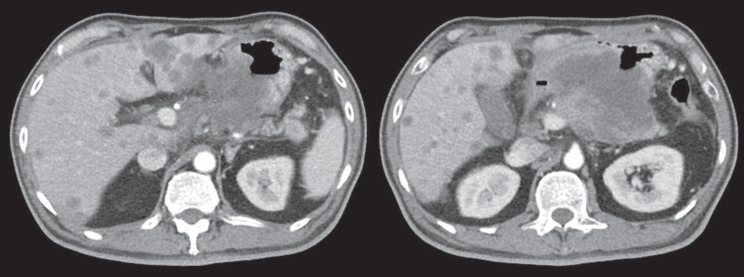
CT scan showing a heterogenous enhanced gastric mass abutting and probably invading the duodenum and pancreas with multiple metastatic lesions in the liver.

Computed tomography (CT) scan showed a huge heterogenous contrast-enhanced mass at the stomach body abutting and probably invading the duodenum and pancreas with numerous nodules in bilateral hepatic lobes and peritoneal cavity, including perigastric region (Figure 1). The picture was reasonably attributed to an advanced gastric cancer with multiple hepatic and peritoneal metastases.

**Figure 2.**
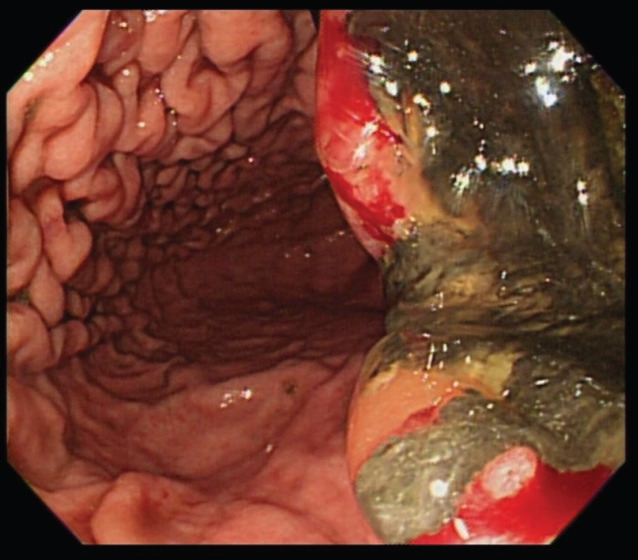
A giant gastric ulcer over lesser curvature of stomach (right half of the figure) revealed by upper gastrointestinal tract endoscopy.

**Figure 3.**
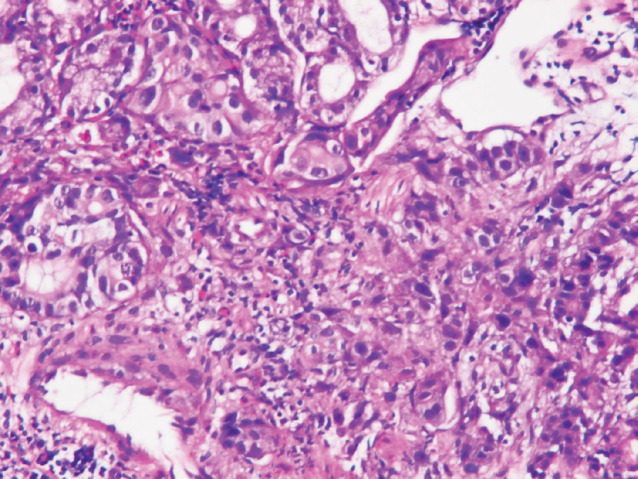
Poorly differentiated carcinoma of stomach, lesser curvature site, endoscopic biopsy (haematoxylin and eosin stain ×400).

**Figure 4.**
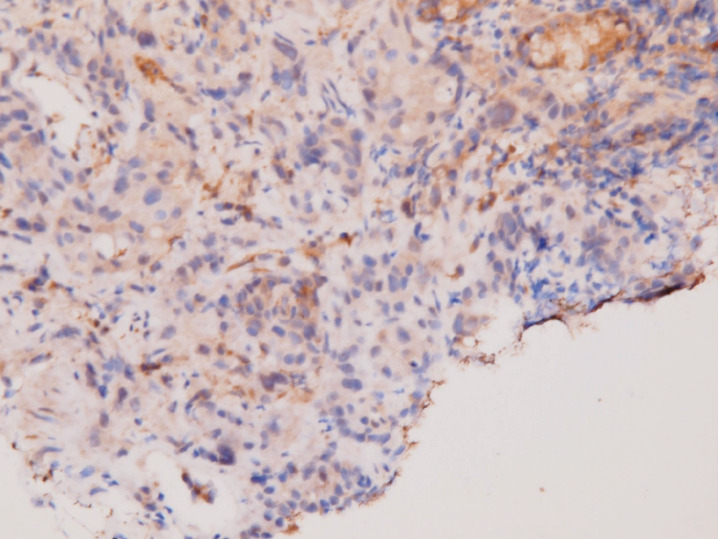
Immunohistochemical staining of DPD. Moderate positivity in tumour cells.

**Figure 5.**
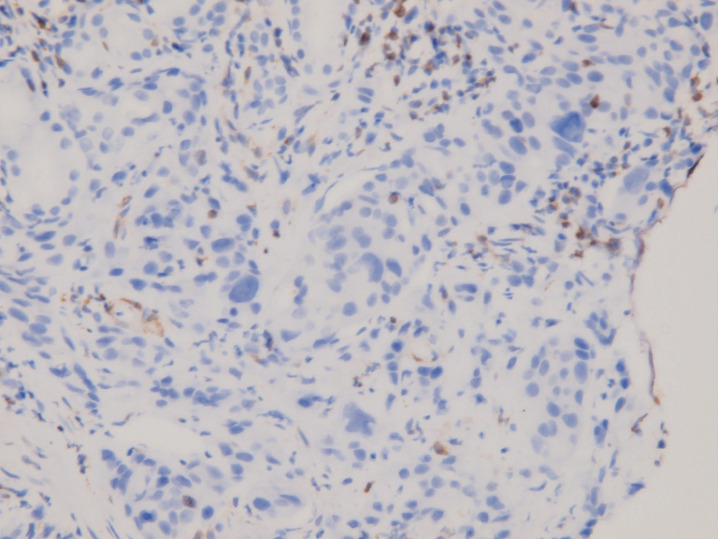
Immunohistochemical staining of Bcl-2. Negative in tumour cells; positive for tumour-infiltrating B-cells.

An upper gastrointestinal tract endoscopy led to the finding of a giant gastric ulcer over lesser curvature of the stomach (Figure 2). Pathology study of the biopsy specimen from the ulcer confirmed the nature of a poorly differentiated carcinoma (Figure 3) positive for cytokeratin AE1/AE3, with morphology patterns in favour of a gastric rather than a pancreatic origin. Immunohistochemical staining afterwards revealed only moderately positive expression of dihydropyrimidine dehydrogenase (DPD) (Rabbit monoclonal DPYD EPR8811: ab134922, Abcam, Cambridge, UK) (Figure 4) and totally negative expression of Bcl-2 (Clone bcl-2/100/D5, Novocastra^TM^ HD, Leica Biosystems, UK) (Figure 5).

The patient was initially reluctant to receive any further image examination and treatment upon knowing the diagnosis. However, he came back to our oncology clinic for helping 18 days later due to rapid downhill progress of performance status. At that time, haemoglobin concentration had dropped to 6.6 g/dL. Thereafter, red blood cells had been transfused to maintain haemoglobin level above 8 g/dl. Notable also were elevated levels of alkaline phosphatase (313 iu/l, normal 32~91), gamma-glutamyl transferase (230 iu/l, normal 7~64), lactate dehydrogenase (428 iu/l, normal 98~192), and total bilirubin (6.12 mg/dL, normal 0~1.3). The renal function was still within normal limits and no evidence of viral hepatitis B and C infection was detected. In spite of a normal value of alpha fetoprotein (3.74 ng/mL, normal 0~9), serum levels of carcinoembryonic antigen (50.99 ng/mL, normal 0~5), cancer antigen 125 (1,894.7 iu/mL, normal 0~35), and cancer antigen 19-9 (78257.6 μ/mL, normal 0~35) were abnormally high.

At this moment, he took our advice and agreed to systemic chemotherapy with a regimen composed of cisplatin 50 mg/m^2^ on day 1, leucovorin 500 mg/m^2^ followed by bolus fluorouracil 500 mg/m^2^ and 22-hour continuous fluorouracil 2,000 mg/m^2^ on day 2 and 3. This protocol, designed to be given on a bimonthly schedule, was modified from those adopted in two previous clinical trials [12, 13]. When calculating the absolute doses for the patient, we used 1 m^2^ as his body surface area in hope of avoiding too severe adverse effects for such a fragile patient.

**Figure 6.**
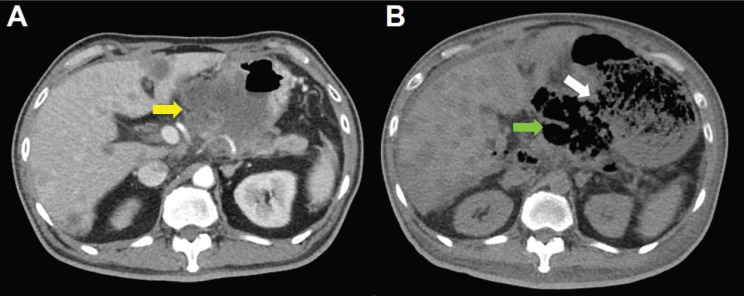
CT scan of the abdomen, horizontal view. A. June 8, 2019. Yellow arrow: the huge gastric tumour. B. July 3, 2019. White arrow: gastric perforation site. Green arrow: Necrotising tumour lysis simulating emphysematous pancreatitis.

**Figure 7.**
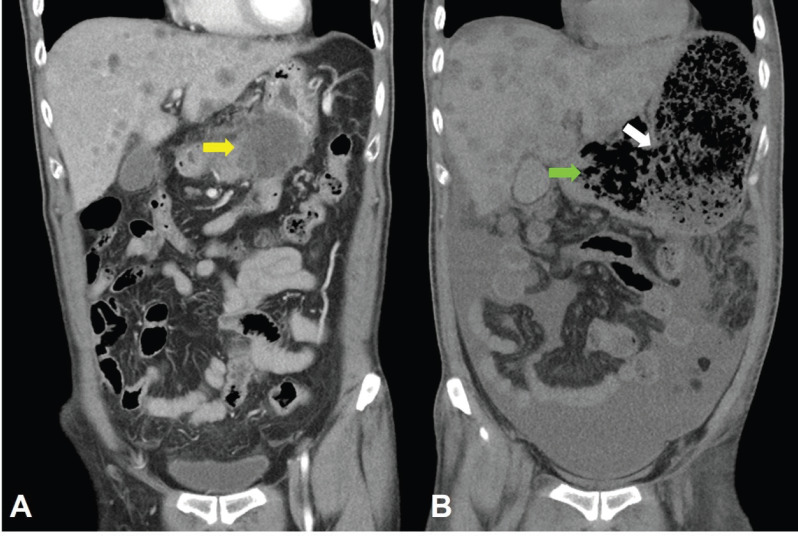
CT scan of the abdomen, coronal view. A. June 8, 2019. Yellow arrow: the huge gastric tumour. B. July 3, 2019. White arrow: gastric perforation site. Green arrow: Necrotising tumour lysis simulating emphysematous pancreatitis.

**Figure 8.**
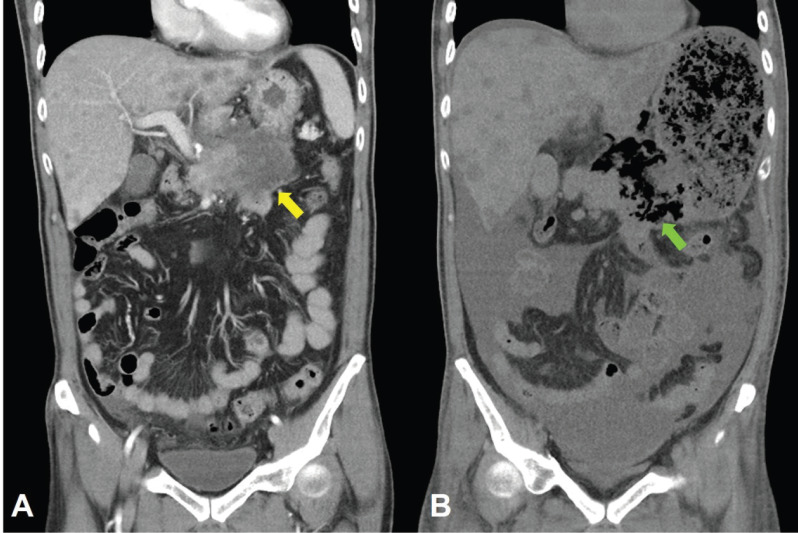
CT scan of the abdomen, coronal view. A. June 8, 2019. Yellow arrow: the huge gastric tumour. B. July 3, 2019. Green arrow: Necrotising tumour lysis simulating emphysematous pancreatitis.

Unfortunately, a catastrophic event did happen 2 days after finishing the first course of chemotherapy. The patient experienced sudden onset of intolerable abdomen pain with hypoactive bowel sounds, muscle guarding, and definitely apparent rebounding pain. An emergent non-contrast CT scan gave a picture of distended stomach fully filled with foods, irregular mottled gas collections between stomach and duodenum, ascites accumulation, and free air in the abdomen cavity, leading to an impression of gastrointestinal tract perforation with the perforation site clearly seen (Figures 6–8). In comparison with CT scan performed at initial diagnosis, it seemed that a severe necrotising tumour lysis induced by chemotherapy, morphologically resembling emphysematous pancreatitis [14–16], could explain the whole scenario logically.

The patient’s family understood the nature of the disease and the dismal prognosis of this subsequent emergent condition very well. Although we planned to sample blood for confirming features characteristic for tumour lysis syndrome, such as serum potassium, uric acid and lactate dehydrogenase levels, they declined further laboratory examination, decided not to let the patient be operated and requested only supportive care with strong pain control. Air hunger with blood oxygen desaturation, drowsy consciousness and blood pressure dropping as the signs of septic shock were observed about 4 hours after abdomen pain attacked. Vital signs became undetectable 2 more hours later.

## Discussion

Clinical outcomes of gastrointestinal perforation in metastatic gastric cancer patients undergoing palliative chemotherapy were very poor, especially for undifferentiated carcinoma and patients with septic shock [17]. Peculiarly, gastric perforation due to severe necrotising tumour lysis of a massive stomach lesion, simulating emphysematous pancreatitis, leading to rapidly progressive septic peritonitis like what was seen in this patient, so far as we know, has been hardly reported in the English literature.

Although the risk is low [18], tumour lysis of gastric cancer, either spontaneous [19, 20] or induced by chemotherapy [21, 22], occurred sporadically. Nevertheless, traditional concern about tumour lysis chiefly focus on life-threatening metabolic complications, including hyperuricemia, hyperkalaemia, hyperphosphatemia and hypocalcaemia, which result in renal failure, cardiac arrhythmia, and neurologic deficit [23, 24]. The patient reported here, on the contrast, faced mainly a rare mechanic complication of tumour lysis syndrome. Accordingly, the underlying causes of this unusual tumour lysis phenomenon deserve careful investigation.

The quantity and activity of cellular DPD, the rate-limiting metabolic enzyme of fluoropyrimidine, are significantly correlated with toxicity of fluropyrimidine [25] and the intratumoural DPD expression level is directly linked to chemosenstivity of fluropyrimidine [26, 27]. Therefore, the not so strong DPD expression in our patient’s tumour specimen might be considered as a possible cause of so severe a tumour lysis on receiving fluorouracil therapy. In support of this view, pharmacogenetic variants of DPD and its deficiency have been suggested to guide individualisation of fluropyrimidine dosage [28] and pre-therapeutic survey of DPD activity was recommended for prevention of fluorouracil-induced early severe toxicity [29].

On the other hand, the apoptosis pathway could also play an important role in determining chemotherapy sensitivity. Bcl-2, an anti-apoptosis protein, has been identified as a hopeful major target in cancer therapy [30, 31]. Increasing Bcl-2 expression in gastric cancer cells led to fluorouracil resistance [32], while interference of Bcl-2 expression made gastric adenocarcinoma cells more sensitive to fluorouracil [33]. Hence, the loss of Bcl-2 in our patient’s gastric cancer most likely contributes to such an extreme necrotising tumour lysis causally.

The current therapeutic approach for stage IV gastric cancer with incurable metastasis aims at volume reduction surgery after adequate chemotherapy probably in combination with targeted or anti-angiogenesis agents [34]. It is exciting to see so many novel therapeutic modalities in development for advanced-stage gastric cancer [35, 36]. Nonetheless, based on our experience in this patient, tumour lysis syndrome with severe adverse complications must be taken into consideration when more and more efficient agents are approved for clinical use. Among them, in our opinion, anti-angiogenic monoclonal antibody ramucirumab, with its potential risk of wound healing impairment and gastrointestinal perforation [37, 38], is an example in case of treating a massive gastric tumour like our patient’s.

## Conclusions

Despite reducing chemotherapy dose in advance, disastrous tumour lysis with gastric perforation still occurred during treatment of this patient’s huge cancer lesion. Based on this painful experience, we recommend that pre-emptive survey of key metabolic enzyme deficiency and other relative molecules like anti-apoptotic Bcl-2 appears be a necessary step in cancer patient receiving fluropyrimidine therapy. In addition to that, well prepared rescue plan including surgical intervention seems mandatory in treating patients with heavy gastrointestinal tumour burdens like ours. Regarding the risk of gastrointestinal tract perforation, how to get a balance between astonishing efficacy and too severe adverse effects remains an issue to be intelligently solved.

## Conflicts of interest

The authors declare that they have no competing interests regarding publication of this case report.

## Funding

There is no financial support for this report.

## Consent for publication

Written informed consent was obtained from the patient’s daughter for publication of this case report and any accompanying images. A copy of the written consent is available for review by the Editor-in-Chief of this journal.
